# Neoadjuvant Immune Checkpoint Inhibitors for Resectable Hepatocellular Carcinoma: A Systematic Review and Meta-Analysis

**DOI:** 10.3390/cancers15030600

**Published:** 2023-01-18

**Authors:** Mei Zhao, Shanwen Chen, Conggui Li, Yingying Du, Ping Li

**Affiliations:** 1 Department of Chinese Integrative Medicine Oncology, The First Affiliated Hospital of Anhui Medical University, Hefei 230032, China; 2 Department of Integrated Traditional Chinese and Western Medicine, Anhui Medical University, Hefei 230032, China; 3 Department of Otorhinolaryngology—Head and Neck Surgery, The First Affiliated Hospital of Anhui Medical University, Hefei 230032, China; 4 Department of Oncology, The First Affiliated Hospital of Anhui Medical University, Hefei 230032, China

**Keywords:** neoadjuvant, ICIs, hepatocellular carcinoma, efficacy, safety

## Abstract

**Simple Summary:**

Approximately 80% of patients with hepatocellular carcinoma (HCC) experience recurrence within five years after surgery. Currently, there is no standard protocol for the application of neoadjuvant therapy in HCC, but neoadjuvant immunotherapy has been shown to influence the survival of patients with other tumors. This systematic review and meta-analysis aimed to assess the reported efficacy and safety of neoadjuvant immune checkpoint inhibitors (ICIs) for resectable HCC. An overview of 9 studies showed neoadjuvant ICIs provide therapeutic benefits in terms of histopathological response in resectable HCC and were well tolerated.

**Abstract:**

Resectable hepatocellular carcinoma (HCC) has poor prognosis because of its high recurrence rate. Immunotherapy has been tried for neoadjuvant therapy as it has shown excellent performance in the treatment of advanced HCC. This systematic review and meta-analysis aimed to assess the reported efficacy and safety of neoadjuvant immune checkpoint inhibitors (ICIs) for resectable HCC. Electronic databases, including PubMed (MEDLINE), Embase, the Cochrane Library, and ClinicalTrials.gov were systematically searched to identify published and ongoing studies evaluating the efficacy and safety of neoadjuvant ICIs for resectable HCC up to October 2022. The odds ratio (OR) and 95% confidence interval (CI) were calculated. Heterogeneity and subgroup analyses were performed, and data quality was assessed. The study was registered with PROSPERO (registration number: CRD42022371495). A total of 193 patients from 9 studies were included in this meta-analysis. The overall pathological complete response (pCR) rate was 12.9% (95%CI, 6.7–19.1%), and major pathological response (MPR) rate was 27.3% (95%CI, 15.1–39.4%), indicating a favorable association with neoadjuvant ICIs (pCR: OR = 0.17, *p* < 0.00001; MPR: OR = 0.38, *p* = 0.001). The pooled OR values for the incidence of grade 3 to 4 treatment-related adverse events and surgical delay rate were 0.26 and 0.05, respectively, which were significantly in favor of neoadjuvant ICIs (*p* < 0.0001; *p* < 0.00001, respectively). The subgroup analyses did not demonstrate superiority of one ICI over another ICI or combination therapy. The present study found that neoadjuvant ICIs were well tolerated by patients with resectable HCC and conferred therapeutic benefits in view of histopathological response results.

## 1. Introduction

Hepatocellular carcinoma (HCC) accounts for approximately 90% of all primary liver cancers and has become the third leading cause of cancer-related mortality worldwide [1,2]. Although several HCC therapies have been developed in the past decades, the 5-year survival rate of HCC patients remains less than 20% [3]. Liver resection (LR) remains the first treatment option for early-stage HCC patients with adequate liver functional reserve, but only approximately 15% of hepatocellular carcinomas are diagnosed early enough to be treated by curative treatments (LR or liver transplantation) [4,5]. However, the long-term survival outcomes for patients with resectable HCC are unsatisfactory, and the risk of 5-year-recurrence after surgery is as high as 80% [6,7]. The rationale behind neoadjuvant therapy is that the early introduction of systemic therapy can potentially decrease the risk of recurrence, remove distant microscopic metastases, and convert unresectable disease into resectable disease [8].

Currently, there is no standard protocol to guide the application of neoadjuvant therapy for patients with HCC, either systemically or topically. Transarterial chemoembolization (TACE), sorafenib, and cytotoxic agents have been investigated as potential neoadjuvant therapies for the treatment of resectable HCC [9,10,11]. However, none of them yield satisfactory survival benefits. Targeting the immune checkpoint programmed cell death protein-1 (PD-1), alone or in combination with CTLA-cytotoxic T-cell antigen 4 (CTLA-4) blockade, in advanced HCC demonstrated a survival benefit [12,13,14,15]. Additionally, a combination of immune checkpoint inhibitors (ICIs) and an anti-angiogenic drug showed superior overall survival compared with sorafenib in first-line therapy [16,17]. The efficacy and safety of neoadjuvant ICIs have been evaluated in non-small cell lung cancer, melanoma, etc. [18,19,20,21]. For example, results from the CheckMate 816 trial demonstrated that neoadjuvant use of nivolumab and standard chemotherapy for lung cancer not only improved the pathological complete response (pCR) rate (24.0% vs. 2.2%, *p* < 0.001), but also extended median event-free survival (EFS) to 31.6 months (20.8 months in the chemotherapy group, *p* = 0.005) [22]. Given the excellent efficacy of immunotherapy in advanced HCC and the success of neoadjuvant immunotherapy in other tumors, investigators hypothesized that perioperative immunotherapy might significantly benefit patients with resectable HCC, and recent studies have corroborated this.

The published retrospective studies and case reports report that neoadjuvant monotherapy or combined immunotherapy before LR or liver transplantation can reduce the recurrence rate and mortality after surgery by achieving complete or partial pathological response [23,24,25,26]. Currently, several prospective clinical trials evaluating the efficacy and safety of neoadjuvant ICIs for resectable HCC are ongoing and have provided promising preliminary results, but all of the relevant studies are phase I or II with small sample sizes. Therefore, based on currently available data, we conducted a meta-analysis to assess the efficacy and safety based on pCR, MPR, TRAEs, and surgical delay rate of neoadjuvant immunotherapy for resectable HCC. To date, this is the first meta-analysis to address this topic for patients with HCC, and we hope to provide an objective and comprehensive evaluation of existing studies, offering a more reliable and stable reference for evaluating whether neoadjuvant immunotherapy can be used in HCC. Given that there is no standard neoadjuvant systemic treatment protocol for HCC, this meta-analysis will provide a theoretical basis for the design of future phase III clinical studies to investigate the benefits of neoadjuvant immunotherapy in HCC patients.

## 2. Materials and Methods

This systematic review followed the Preferred Reporting Items for Systematic Reviews and Meta-analyses (PRISMA) guidelines and checklists [27]. No approval was required from the institutional ethics review board because this article did not involve any individual patient data. The study was registered in the International prospective register of systematic reviews (PROSPERO) with the unique identification number CRD42022371495.

### 2.1. Search Strategy

We performed a systematic search of PubMed (MEDLINE), Embase, the Cochrane Library, and ClinicalTrials.gov to retrieve studies investigating the use of neoadjuvant immunotherapy in HCC published before 1 October 2022. Additionally, we searched for ongoing clinical trials on neoadjuvant immunotherapy in the management of HCC that were presented at international oncology conferences. Medical subject terms such as hepatocellular carcinoma, neoadjuvant therapy, and immunotherapy were used to conduct the search. Please refer to the Supplementary methods for the detailed search strategy.

### 2.2. Study Selection

All publications that met the following criteria were included: (1) The clinical trial included patients with resectable HCC; (2) Immune checkpoint inhibitors were used as neoadjuvant therapy; (3) The study reported at least one of the following primary outcomes: pathological complete response (pCR) defined as no viable tumor cells, major pathological response (MPR) defined as less than 10% residual viable tumor cells in the resected tumor, treatment-related adverse events (TRAEs) or grade 3–4 TRAEs, and surgical delay rate. Publications that met one of the following criteria were excluded: (1) Patients had unresectable primary or metastatic disease; (2) The number of included patients was less than 10; (3) The research outcome did not meet our specified outcomes; (4) Lack of valid or adequate data for assessing the efficacy and safety of neoadjuvant immunotherapy; (5) Repeated publications; (6) Reviews, meta-analyses, case reports, or case series.

### 2.3. Data Extraction

Two investigators (M.Z. and S.C.) independently identified and extracted articles for possible inclusion. Any discrepancies were resolved by submission to a third reviewer (P.L.). The full text of the identified articles was retrieved and analyzed. For each study, the following data were recorded: first author, year of publication, clinical trial, NCT number, intervention model, masking, study type, study phase, location, article type, main inclusion criteria, ICI drug, sample size, pCR, MPR, incidence of TRAEs or grade 3–4 TRAEs, and surgical delay rate.

### 2.4. Quality Assessment and Risk of Bias

The selected studies were assessed for risk of bias using the assessment tool recommended by Cochrane Handbook 5.1.0, which includes the following: (1) random sequence generation; (2) concealment of allocation; (3) blinding of participants and personnel; (4) blinding of outcome assessment; (5) completeness of outcome data; (6) selective reporting of outcomes; and (7) other bias. Two reviewers (M.Z. and S.C.) independently assessed the risk of bias. Disagreements were resolved through discussion or referral to a third reviewer (P.L.)

### 2.5. Data Analysis

This meta-analysis was performed using non-comparative binary data in RevMan software version 5.3 (Cochrane Collaboration) because the majority of the included studies were single-arm clinical trials. The effect index (P) and its standard error SE (P) for uncontrolled binary data were calculated using the following formula: P = ln(odds) = ln (X/(n − X)); SE (P) = SE (ln(odds)) = √ (1/X + 1/(n − X)). The effect measures were the odds ratio (OR) and 95% confidence interval (CI) [28,29]. OR < 1 implied that neoadjuvant immunotherapy had a therapeutic advantage. Heterogeneity of the results across studies was determined based on the heterogeneity index (I^2^); if the heterogeneity was significant (*p* < 0.1) or greater than 50% (≥50%), a random-effect model was used; if insignificant (*p* ≥ 0.1) and lower than 50% (<50%), a fixed effect model was adopted. Subgroup analyses were performed on specific immune checkpoint inhibitors or in combination with other treatments. *p* < 0.05 was considered to be statistically significant.

## 3. Results

**Figure 1:**
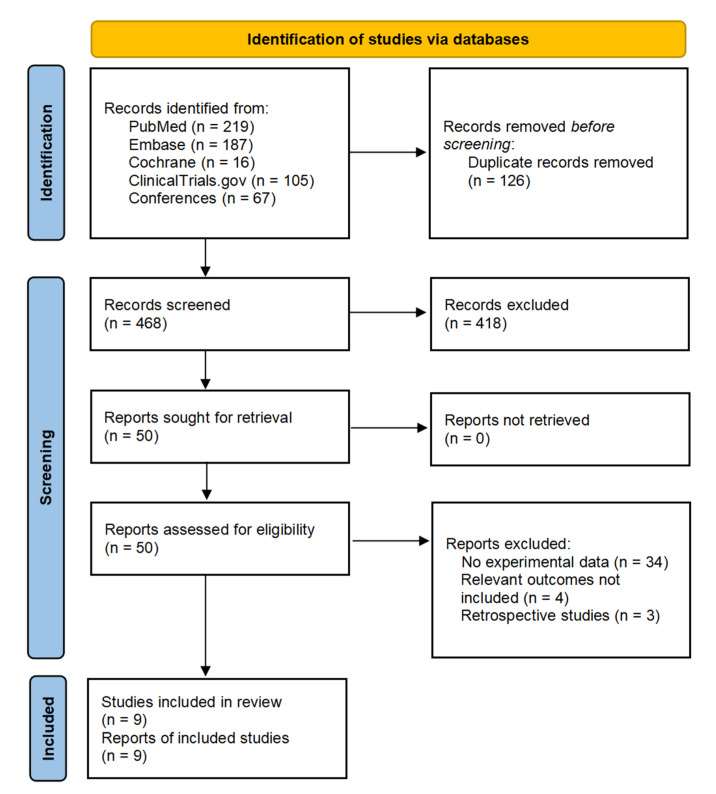
Flowchart of study selection.

We identified 594 citations based on the search strategy. After deleting duplicates, screening titles, and abstracts, and reviewing the available full texts, 9 studies with a total of 193 patients met the inclusion criteria and were included in the meta-analysis. The detailed strategy for study selection is shown in Figure 1. Five included studies were ongoing trials for which only the abstracts were available, while the remaining four studies were published as full texts. Table 1 provides a summary of the characteristics of the included studies [30,31,32,33,34,35,36,37,38]. Additionally, Supplementary Table S1 provides information on other ongoing clinical trials of neoadjuvant immune checkpoint inhibitors.

### 3.1. Efficacy of Neoadjuvant Immune Checkpoint Inhibitors

**Figure 2:**
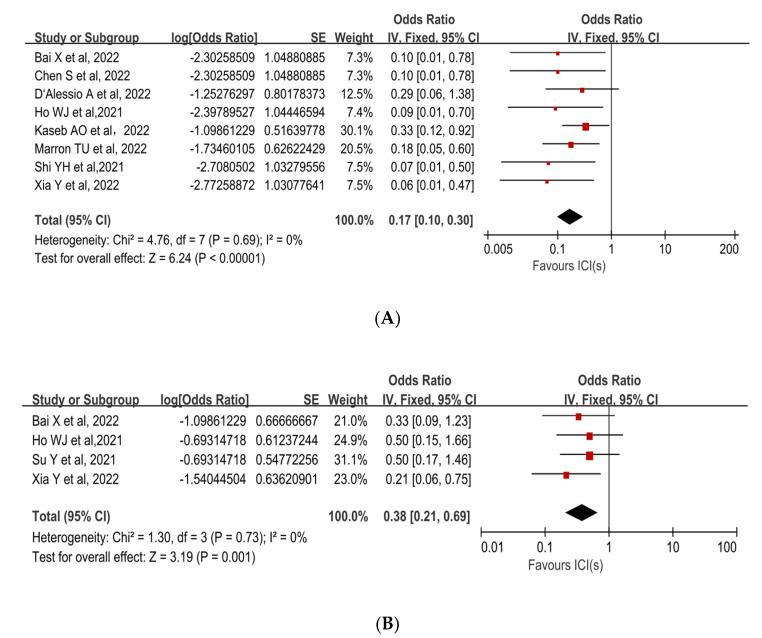
Forest plot of the efficacy of neoadjuvant immune checkpoint inhibitors in resectable hepatocellular carcinoma. (**A**) pCR, (**B**) MPR. pCR: pathological complete response; MPR: major pathological response [30,31,32,33,34,35,36,37,38].

pCR was reported in 8 studies with rates ranging from 5.9% to 25%. The mean pCR rate was 12.9% (95%CI, 6.7–19.1%). The pooled results of the included trials demonstrated a statistically significant benefit of using neoadjuvant ICIs (OR, 0.17; 95%CI, 0.10–0.30; *p <* 0.00001; Figure 2A [30,32,33,34,35,36,37,38]). Due to the low degree of heterogeneity in the results, a fixed-effect model was adopted (*p* = 0.69, I^2^ = 0%).

The MPR to neoadjuvant ICIs was reported in three studies. The mean MPR rate was 27.3% (95%CI, 15.1–39.4%), with a range of 17.6% to 33.3%. Individual ORs for each eligible study in terms of MPR were in favor of neoadjuvant ICIs (individual OR *<* 1.0). The combined OR was 0.38 (95%CI, 0.21–0.69) with a statistically significant difference (*p* = 0.001), indicating that neoadjuvant ICIs were beneficial (Figure 2B [31,32,34,38]). Because there was no significant heterogeneity among the studies (*p* = 0.73, I^2^ = 0%), a fixed-effect model was adopted.

### 3.2. Safety of Neoadjuvant Immune Checkpoint Inhibitors

**Figure 3:**
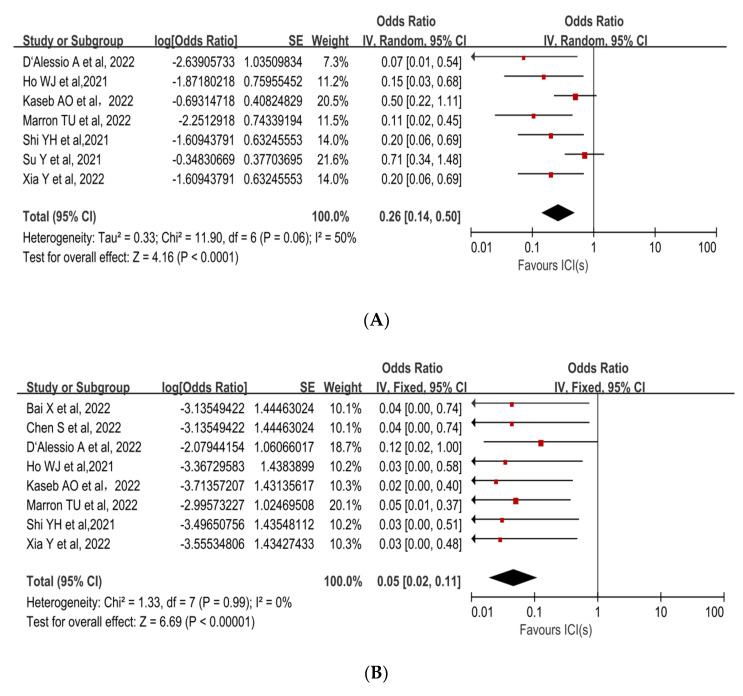
Forest plot of the safety of neoadjuvant immune checkpoint inhibitors in resectable hepatocellular carcinoma. (**A**) Grade 3–4 TRAEs, (**B**) Surgical delay rate. ICIs: immune checkpoint inhibitors. TRAEs: treatment-related adverse events [30,31,32,33,34,35,36,37,38].

The incidence of treatment-related adverse events (TRAEs), as defined by the National Cancer Institute’s Common Terminology Criteria for Adverse Events (CTCAE) version 4.0, is associated with the safety of neoadjuvant ICIs. No patients died as a result of TRAEs in any of the trials. The incidence of preoperative grade 3–4 TRAEs was 22.4% (95%CI, 15.5–29.3%), and included pneumonitis, hepatitis, pruritus, maculopapular rash, myasthenia gravis, infection, lipase increase, and leukocyte reduction. A pooled analysis of seven studies revealed an OR of 0.26 (95%CI, 0.14–0.50) and an acceptable safety profile for neoadjuvant ICIs (*p* < 0.0001, Figure 3A [30,31,32,33,34,35,37]). The sensitivity analysis was performed because there was a high level of heterogeneity (*p* = 0.06, I^2^ =50%). The Su Y et al. study [31], which had the highest weight in the analysis, was excluded, and the heterogeneity decreased (Supplementary Table S2).

Surgical delay rate was defined as the ratio of patients whose surgery was delayed due to adverse events caused by neoadjuvant ICIs to all patients expected to have surgery. The mean surgical delay rate was 1.7% (95%CI, 0–4.1%). Among all patients who underwent surgical resection, one patient had a 2-week delay in surgery due to grade 3 pneumonitis and another patient had a surgery delay due to deterioration of liver function, which was not related to ICI treatment. The pooled OR (0.05,95% CI, 0.02–0.11) was in favor of neoadjuvant immunotherapy (*p* < 0.00001, Figure 3B [30,32,33,34,35,36,37,38]).

### 3.3. Subgroup Analyses

**Figure 4:**
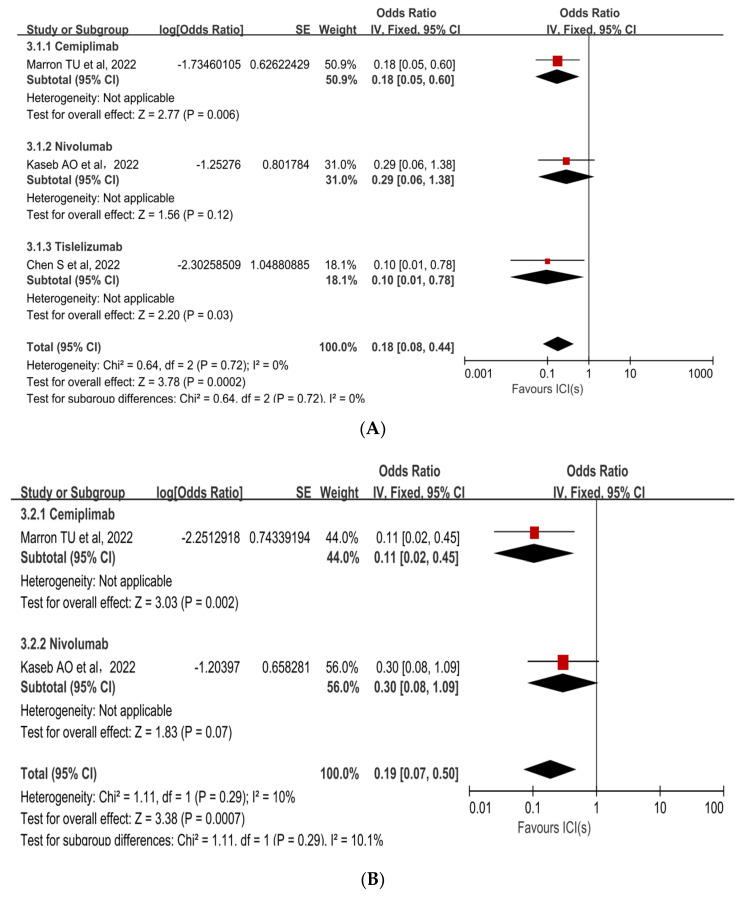
Subgroup analysis of immune checkpoint inhibitor drug types for (**A**) pCR and (**B**) Grade 3−4 TRAEs. ICI: immune checkpoint inhibitor; pCR: pathological complete response; TRAEs: treatment-related adverse events [33,35,36].

**Figure 5:**
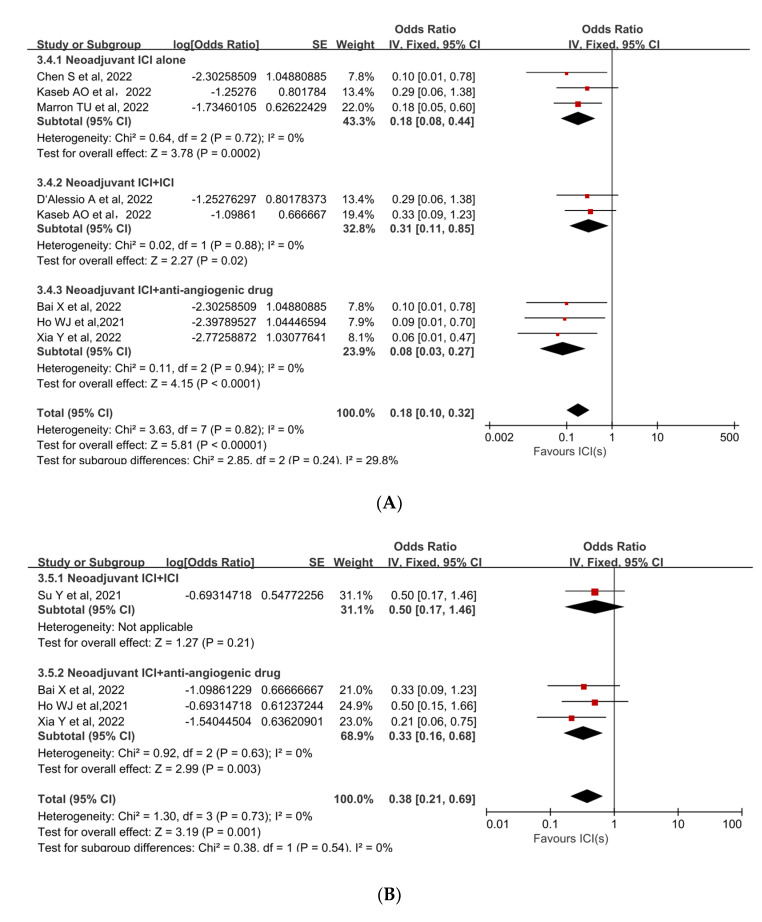

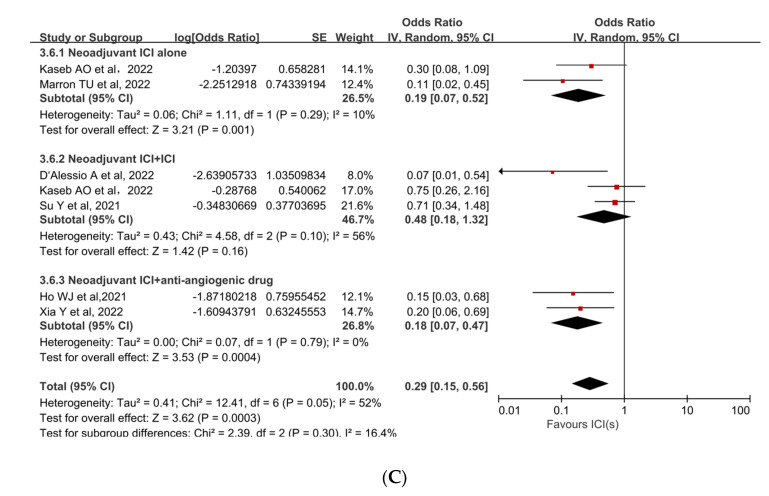
Subgroup analyses based on neoadjuvant immune checkpoint inhibitor combinations for (**A**) pCR, (**B**) MPR, and (**C**) Grade 3−4 TRAEs. ICI: immune checkpoint inhibitor; pCR: pathological complete response; MPR: major pathological response; TRAEs: treatment-related adverse events [31,32,33,34,35,36,37,38].

Subgroup analyses were performed to ascertain the possible sources of heterogeneity and to clarify differences in efficacy and safety between different types of ICIs and different combinations of ICIs. Subgroup analysis revealed that no single ICI was superior to another (Figure 4A [33,35,36], Figure 4B [33,35]). Similarly, subgroup analyses of safety and efficacy outcomes (pCR, MPR, and Grade 3−4 TRAEs) revealed no differences between single ICIs or combinations of ICIs and antiangiogenic drugs (Figure 5A [32,33,34,35,36,37,38], Figure 5B [31,32,34,38], Figure 5C [31,32,33,34,35,37]). In the subgroup analysis of grade 3 to 4 TRAEs, high heterogeneity was detected in the study with combined ICI + ICI treatment (Su, Y. et al., 2022 [31]), which contributed the most to the heterogeneity in the overall results.

### 3.4. Risk of Bias

The review authors’ judgments of the risk of bias for each item are presented in Supplementary Figures S1 and S2.

## 4. Discussion

This meta-analysis of efficacy (pCR, MPR) and safety (Grade 3–4 TRAEs, surgical delay rate) initially demonstrated that neoadjuvant immune checkpoint inhibitors may have therapeutic advantages in terms of histopathological response and acceptable toxicity profiles in patients with resectable HCC.

The mean pCR and MPR rates in our meta-analysis were 12.9% (95%CI, 6.7–19.1%) and 27.3% (95%CI, 15.1–39.4%), respectively, with a maximum pCR of 25% (reported by Kaseb, A.O. and colleagues [35]) and a maximum MPR of 33.3% (reported by Su, Y. et al. and Ho, W.J. et al. [31,32]). The two studies conducted by Su, Y. et al. and Kaseb, A.O. et al. reported progression free survival (PFS) of 13.4 months (95%CI, 1.4—not reached), 9.4 months (1.47—not estimable [NE]) with nivolumab, and 19.53 months (2.33—NE) with nivolumab plus ipilimumab, respectively [31,35]. No study reported overall survival (OS) because the studies were still in the process of following up. Therefore, it is not clear whether a significant pathologic response was associated with improved prognosis due to insufficient follow-up time. The studies by Marron, T.U. et al. and Kaseb, A.O. et al. showed that patients with 50% or more necrosis had increased density of immune infiltration and a greater number of tumor-infiltrating lymphocytes compared with those with little or no necrosis [33,35]. Kaseb, A.O. and colleagues found that 6 patients who showed a major pathologic response to neoadjuvant immune checkpoint therapy did not experience recurrence after a median follow-up of 26.8 months; in comparison, 7 of the 14 patients who did not have a major pathologic response experienced recurrence [35]. Despite the small sample size, there were differences in the recurrence-free survival between patients who had a major pathologic response and those who did not. Ho WJ and colleagues found a statistically significant long-term disease-free survival (DFS) in patients who achieved a major pathological response, with DFS intervals of more than 230 days [32]. Although this meta-analysis demonstrated the histopathological response results of neoadjuvant immunotherapy, the long-term survival efficacy of this therapy remains unknown.

Neoadjuvant immunotherapy has proven to be feasible in other malignancies. Additionally, patients who achieve a significant pathological response to neoadjuvant ICIs have improved survival after surgery [22,39,40,41]. It has been reported that tumor antigens present before LR may enable neoadjuvant ICIs to generate stronger and longer-lasting antitumor T-cell immune responses than the adjuvant setting, making it more effective against micro-metastases, which are thought to be associated with HCC recurrence [8,42,43]. Several studies have concluded that neoadjuvant ICIs have improved efficacy in eradicating metastatic disease compared to adjuvant ICIs [44], and preclinical models have confirmed this view. The investigators observed longer survival and enhanced activation of tumor-specific CD8+ T cells in mice administered PD-1 blockade preoperatively compared to mice receiving only postoperative treatment [45]. The reasons might be as follows: 1. Untreated resectable HCC patients have a potent immune system, and ICI can cause robust immune responses that also exist post-operatively [8]. 2. Preoperative use of immunotherapy could initiate T-cell responses to tumor neoantigens, whereas in adjuvant therapy, the only remaining neoantigens were from micro-metastases, which may translate into less immune initiation and activation [19,46]. 3. Disruption of the immune system due to surgery leaves patients in a state of immunosuppression, hindering activation of T cells and potentially further limiting the efficacy of adjuvant ICIs [47,48,49].

In this study, the mean incidence of grade 3–4 TRAEs in 5 studies was 22.4% (95%CI, 15.5–29.3%), which was consistent with previously reported data in advanced HCC [12,13,14,15,16,17], with a maximum of 41.4% in the study by Su, Y. and colleagues [31]. The intended primary endpoints of safety and tolerability were achieved in all four completed studies, which supported further studies to investigate the efficacy of these regimens. Safety results showed that the neoadjuvant immunotherapy did not increase the difficulty and risk of surgery [32,33,35]. Xia, Y. et al. found that the amount of bleeding during LR and the duration of LR increased after neoadjuvant camrelizumab + apatinib treatment, which was related to the application of an anti-angiogenic drug, although they had stopped using apatinib 3 weeks before surgery [34]. Therefore, more adequate preparation and professional operation are needed in the perioperative period to reduce complications. On the other hand, a limitation that must be considered is the need to delay surgery with curative intent due to the risk of significant toxicities. In the present study, only one case of TRAEs resulted in delayed surgery due to neoadjuvant immunotherapy. Although the patient developed grade 3 pneumonitis and required steroids in neoadjuvant cemiplimab therapy, causing a two-week delay in surgery, he eventually underwent successful surgical resection [33].

Subgroup analyses in this study revealed no benefit of combined ICIs or ICIs in combination with anti-angiogenic drugs compared with monotherapy. There were no statistically significant differences in efficacy and safety across the various ICIs. Clinical trials of atezolizumab + bevacizumab, pembrolizumab + lenvatinib, and other ICIs and anti-angiogenic therapy combinations have demonstrated the superiority of combination therapy in patients with unresectable HCC [16,50,51]. Angiogenic factors and their receptors contribute to the formation of an immunosuppressive tumor microenvironment by acting directly on innate and adaptive immune cells and indirectly on endothelial cells [52,53,54]. Anti-angiogenic therapy alleviates these immunosuppressive effects by increasing tumor infiltration of mature dendritic cells and effector T cells, which reduces tumor infiltration by immunosuppressive cells such as regulatory T cells and myeloid-derived suppressor cells. Anti-angiogenic therapy combined with immunotherapy has been shown to promote vascular normalization, alleviate vascular endothelial growth factor (VEGF)-mediated immunosuppression, and enhance anti-tumor immune responses in patients with HCC and other malignancies [52,55,56,57]. It was reported that treatment with cabozantinib, a multi-kinase inhibitor with anti-vascular effect, alone was associated with systemically and locally enhanced pro-immune responses and promoted T-cell differentiation towards less depleted phenotypes. Cabozantinib elicits immune responses that potentiate the effects of nivolumab as an immune-mediated therapeutic synergy [32]. The two studies included in this review (Ho, W.J. et al., 2021 [32] and Xia, Y. et al., 2022 [34]) used nivolumab + cabozantinib and camrelizumab + apatinib, respectively, with mean pCR and MPR rates of 6.9% (95%CI, 0–16.7%) and 24.1% (95%CI, 7.6–40.7%), respectively. Although the results of the subgroup analyses did not show any histopathological response advantage for the combined use of ICIs and anti-angiogenic therapy, further studies are needed to determine their long-term survival benefit.

Exploring treatment-related biomarkers is one of the important purposes of neoadjuvant therapy clinical trials. PD-L1 expression status has been used as a biomarker of treatment response in some malignancies, such as NSCLC and bladder cancer, while its use for guiding therapy in liver cancer remains controversial [58]. The predictive role of PD-L1 expression status for outcomes of neoadjuvant immunotherapy is not unknown. Results of the CheckMate 816 clinical trial showed that neoadjuvant nivolumab+ chemotherapy prolonged EFS and increased the pCR rate of NSCLC patients in the subgroup with PD-L1 expression level ≥1% (EFS:21.1 months vs. 18.4 months; pCR:32.6% vs. 16.7%) [22]. This demonstrated that the PD-L1 expression status may be an optional biomarker for neoadjuvant immunotherapy in NSCLC. Unfortunately, the relationship between PD-L1 expression status and efficacy was not clearly defined in any of the nine studies included in this analysis. These phase II studies attempted to explore new biomarkers of neoadjuvant immunotherapy outcomes in HCC. The study by Kaseb AO and colleagues identified an increased percentage of T cells and B cells and an increased ratio of CD8+ T cell/Treg (regulatory T cell) in the tumor microenvironment in patients who developed a significant pathological response compared to those who did not [35]. Ho WJ et al. observed enhanced B-cell infiltration, increased TNF-α expression, CD138+ plasma cell infiltration, and tertiary lymphoid structures (TLS) composed of B cells and T cells in pathological responders, suggesting that B cells are involved in antitumor immunity. Combined with the results of previous studies [59,60,61], these authors considered that tumor-infiltrating B cells may be an important biomarker of antitumor immune response. Certainly, further studies are needed to validate the predictive value of the identified biomarkers.

This meta-analysis had certain limitations. Half of the data were obtained from conference abstracts. Variabilities in study design, treatment regimens, inconsistent etiology of hepatocellular carcinoma, types of immune checkpoint inhibitors, and patient characteristics contributed to heterogeneity, thus limiting the strength of these findings. Additionally, subgroup analysis of PD-L1 expression status was not be performed due to the lack of data, and the effect of PD-L1 level on outcomes cannot be ignored. Lastly, long-term outcomes, such as recurrence-free survival (RFS), disease-free survival (DFS), and overall survival (OS), which can provide a more accurate indication of treatment efficacy, were not reported.

## 5. Conclusions

Our findings suggest that, based on the results of existing studies, neoadjuvant immune checkpoint inhibitors provide therapeutic benefits in terms of histopathological response in resectable HCC and were well tolerated. Conclusive evidence awaits more data from long-term, large-scale clinical trials investigating neoadjuvant immunotherapy in patients with resectable HCC.

## Figures and Tables

**Table 1:** Characteristics of neoadjuvant immunotherapy studies in patients with hepatocellular carcinoma. N/A, not applicable; HCC, hepatocellular carcinoma; NI, neoadjuvant immunotherapy; ICIs, immune checkpoint inhibitor.

Source (Author/Year)	Trial Indentifier	Region	Sample Size	Study Phase	Intervention Model	Masking	Study Type	Randomization Method	Main Inclusion Criteria	Article Type	Neoadjuvant Immuotherapy	Cycles of NI	ICIs Post-Surgery	pCR	MPR	Grade3–4 TRAEs	Surgical Delay
Shi, Y.H. et al., 2021 [30]	NCT03867370	China	18	1b/2	Sequential Assignment	Open Label	Clinical Trial	Randomized	Surgically resectable; has not received local treatment	Conference abstract	Toripalimab (n = 14) or Toripalimab+ Lenvatinib (n = 4)	1	Yes	6.3% (1/16)	NA	16.7% (3/18)	0% (0/16)
Su, Y. et al., 2021 [31]	NCT03510871	China	29	2	Single Group Assignment	Open Label	Clinical Trial	N/A	Potentially eligible for curative surgery (AJCC T3/T2)	Conference abstract	Nivolumab+ ipilimumab	2/4	N/A	NA	33.3%(5/15)	41.4% (12/29)	NA
Ho, W.J. et al., 2021 [32]	NCT03299946	USA	15	1	Single Group Assignment	Open Label	Clinical Trial	N/A	Locally advanced/borderline resectable; high-risk tumor features	Full text	Nivolumab+ Cabozantinib	4	N/A	8.3% (1/12)	33.3% (4/12)	13.3% (2/15)	0% (0/14)
Marron, T.U. et al., 2022 [33]	NCT03916627	USA	21	2	Single Group Assignment	Open Label	Clinical Trial	N/A	Surgical candidate for resection	Full text	Cemiplimab	2	Yes	15% (3/20)	NA	10% (2/21)	5.8% (1/21)
Xia, Y. et al., 2022 [34]	NCT04297202	China	20	2	Single Group Assignment	Open Label	Clinical Trial	N/A	Systemic treatment-naive resectable HCC in intermediate/advanced stage.	Full text	Camrelizumab+ Apatinib	3	Yes	5.9% (1/17)	17.6%(3/17)	16.7% (3/18)	0% (0/17)
Kaseb, A.O. et al., 2022 [35]	NCT03222076	USA	30	2	Parallel Assignment	Open Label	Clinical Trial	Randomized	Patients with HCC who are eligible for surgical resection	Full text	Nivolumab (n = 13) or Nivolumab +Ipilimumab (n = 14)	3	Yes	25% (5/20)	NA	33.3% (9/27)	0% (0/20)
Chen, S. et al., 2022 [36]	NCT04615143	China	11	2	Sequential Assignment	Open Label	Clinical Trial	Non-Randomized	Resectable recurrent HCC after curative ablation	Conference abstract	Tislelizumab	2	Yes	9.1% (1/11)	NA	NA	0% (0/11)
D’Alessio, A. et al., 2022 [37]	NCT03682276	UK	17	1b	Single Group Assignment	Open Label	Clinical Trial	N/A	HCC medically fit to undergo surgery; ineligible for liver transplantation	Conference abstract	Nivolumab+ Ipilimumab	2	N/A	22% (2/9)	NA	7% (1/15)	11% (1/9)
Bai, X. et al., 2022 [38]	NCT04930315	China	32	2	Parallel Assignment	Open Label	Clinical Trial	Randomized	BCLC stage B/C, or CNLC stage was IIa-IIIb, technically resectable	Conference abstract	Camrelizumab+ Apatinib (n = 16)	4	Yes	9.1% (1/11)	27.3%(3/11)	NA	0% (0/11)

## Data Availability

Data for this study is available, as all included articles in this meta-analysis are publicly accessible through PubMed, EMBASE, The Cochrane Library, and ClinicalTrial.gov.

## Supplementary Materials

The following supporting information can be downloaded at: https://www.mdpi.com/article/10.3390/cancers15030600/s1, Supplementary methods: Search strategy for Cochrane/PubMed (MEDLINE)/Embase/ClinicalTrials.gov. Figure S1: Risk of bias graph: review authors’ judgments about the risk of bias of each item, presented as percentages across all included studies; Figure S2: Risk of bias summary: review authors’ judgments about the risk of bias for each item included study; Table S1: Ongoing clinical trials of neoadjuvant immune checkpoint inhibitors in hepatocellular carcinoma; Table S2: Sensitivity analysis for Grade 3–4 TRAEs outcomes.
