# Supplementary material for: Neoadjuvant Immune Checkpoint Inhibitors for Resectable Hepatocellular Carcinoma: A Systematic Review and Meta-Analysis
Source: Cancers (Basel). 2023 Jan 18;15(3):600. doi: 10.3390/cancers15030600 (PMC9913451; doi:10.3390/cancers15030600)
Supplement: Supplementary file 1 [file cancers-15-00600-s001.zip › cancers-2093640-supplementary.pdf]

Supplementary material

sMethods. Supplementary methods

Table S1. Ongoing clinical trials of neoadjuvant immune checkpoint inhibitors in hepatocellular carcinoma

Table S2. Sensitivity analysis for Grade 3-4 TRAEs outcomes

Figure S1. Risk of bias graph: review authors' judgment about each risk of bias item presented as percentages across all included studies.

Figure S2. Risk of bias summary: review authors' judgments about each risk of bias item for each included study.

sMethods. Supplementary methods

Search strategy for Cochrane database:

#1 MeSH descriptor: [Carcinoma, Hepatocellular] explode all trees

#2 (“hepatic cancer”):ti,ab,kw OR (“hepatocellular carcinoma”):ti,ab,kw OR (HCC):ti,ab,kw OR (“liver cancer”):ti,ab,kw OR (“liver carcinoma”):ti,ab,kw (Word variations have been searched)

#3 #1 OR #2

#4 MeSH descriptor: [Antineoplastic Agents, Immunological] explode all trees

#5 MeSH descriptor: [Immune Checkpoint Inhibitors] explode all trees

#6 MeSH descriptor: [Immunologic Factors] explode all trees

#7 MeSH descriptor: [Antibodies, Monoclonal] explode all trees

#8 MeSH descriptor: [Nivolumab] explode all trees

#9 MeSH descriptor: [Ipilimumab] explode all trees

#10 ("immunotherapy*"):ti,ab,kw OR ("immune therap*"):ti,ab,kw OR ("immune treat*"):ti,ab,kw OR ("checkpoint inhibitor*"):ti,ab,kw OR ("PD 1"):ti,ab,kw (Word variations have been searched)

#11 ("Nivolumab"):ti,ab,kw OR ("OPDIVO"):ti,ab,kw OR ("Pembrolizumab"):ti,ab,kw OR ("lambrolizumab"):ti,ab,kw OR ("Keytruda"):ti,ab,kw (Word variations have been searched)

#12 ("Camrelizumab"):ti,ab,kw OR ("Toripalimab"):ti,ab,kw OR ("Sintilimab"):ti,ab,kw OR ("Tislelizumab"):ti,ab,kw OR ("PD L1"):ti,ab,kw (Word variations have been searched)

#13 ("Atezolizumab"):ti,ab,kw OR ("durvalumab"):ti,ab,kw OR ("Cemiplimab"):ti,ab,kw OR ("avelumab"):ti,ab,kw OR ("Ipilimumab"):ti,ab,kw (Word variations have been searched)

#14 ("Tremelimumab"):ti,ab,kw OR ("ctla 4"):ti,ab,kw (Word variations have been searched)

#15 #4 OR #5 OR #6 OR #7 OR #8 OR #9 OR #10 OR #11 OR #12 OR #13 OR #14

#16 MeSH descriptor: [Neoadjuvant Therapy] explode all trees

#17 ("neoadjuvant therap*"):ti,ab,kw OR ("neoadjuvant treatmen*"):ti,ab,kw

#18 #16 OR #17

#19 #3 AND #15 AND #18

Search strategy for Pubmed (MEDLINE):

((((("Neoadjuvant Therapy"[Mesh])) OR ("neoadjuvant treat*"[Text Word])) OR ("neoadjuvant therap*"[Text Word])) AND (((((((((("Antineoplastic Agents, Immunological"[Mesh]) OR "Immunologic Factors"[Mesh]) OR "Immunotherapy"[Mesh]) OR "Immune Checkpoint Inhibitors"[Mesh]) OR "Immunoglobulins"[Mesh]) OR "Programmed Cell Death 1 Receptor"[Mesh]) OR "CD274 protein, human" [Supplementary Concept]) OR "Nivolumab"[Mesh]) OR "Ipilimumab"[Mesh]) OR (("immunotherapy*"[Text Word] OR "immune therap*"[Text Word] OR "immune treat*"[Text Word] OR "checkpoint

inhibitor*"[Text Word] OR "PD 1"[Text Word] OR "Nivolumab"[Text Word] OR "OPDIVO"[Text Word] OR "Pembrolizumab"[Text Word] OR "lambrolizumab"[Text Word] OR "Keytruda"[Text Word] OR "Camrelizumab"[Text Word] OR "Toripalimab"[Text Word] OR "Sintilimab"[Text Word] OR "Tislelizumab"[Text Word] OR "PD L1"[Text Word] OR "Atezolizumab"[Text Word] OR "durvalumab"[Text Word] OR "Cemiplimab"[Text Word] OR "avelumab"[Text Word] OR "Ipilimumab"[Text Word] OR "Tremelimumab"[Text Word]) OR "ctla 4"[Text Word])))) AND (("Liver Neoplasms"[Mesh]) OR ((((("hepatic cancer"[Text Word]) OR ("hepatocellular carcinoma"[Text Word])) OR ("liver cancer"[Text Word])) OR ("liver carcinoma"[Text Word])) OR ("HCC"[Text Word])))

Search strategy for Embase:

#1'neoadjuvant therapy'/exp

#2'neoadjuvant therap*':ti,ab,kw OR 'neoadjuvant treatmen*':ti,ab,kw

#3 #1 OR #2

#4'immunological antineoplastic agent'/exp OR 'immunologic factor'/exp OR 'immunotherapy'/exp OR 'immune checkpoint inhibitor'/exp OR 'immunoglobulin'/exp OR 'programmed death 1 receptor'/exp OR 'programmed cell death ligand 1'/exp OR 'antineoplastic monoclonal antibody'/exp

#5'immunotherap*':ab,ti,kw OR 'immune therap*':ab,ti,kw OR 'immune treat*':ab,ti,kw OR 'checkpoint inhibitor*':ab,ti,kw OR 'ctla-4':ab,ti,kw OR 'pd-1':ab,ti,kw OR 'pd-l1':ab,ti,kw OR 'nivolumab':ab,ti,kw OR 'opdivo':ab,ti,kw OR 'pembrolizumab':ab,ti,kw OR 'lambrolizumab':ab,ti,kw OR 'keytruda':ab,ti,kw OR

camrelizumab:ab,ti,kw OR toripalimab:ab,ti,kw OR sintilimab:ab,ti,kw OR tislelizumab:ab,ti,kw OR cemiplimab:ab,ti,kw OR atezolizumab:ab,ti,kw OR durvalumab:ab,ti,kw OR avelumab:ab,ti,kw OR ipilimumab:ab,ti,kw OR tremelimumab:ab,ti,kw

#6 #4 OR #5

#7 'liver cell carcinoma'/exp

#8 'hepatic cancer':ti,ab,kw OR 'hepatocellular carcinoma':ti,ab,kw OR 'liver cancer':ti,ab,kw OR 'liver carcinoma':ti,ab,kw OR 'hcc':ti,ab,kw

#9 #7 OR #8

#10 #3 AND #6 AND #9

Search strategy for ClinicalTrials.gov:

("Liver Neoplasms" OR "Hepatic Cancer" OR "Hepatocellular Carcinoma" OR "Liver Cancer" OR "HCC") AND (“Neoadjuvant Therapy”)

Table S1. Ongoing clinical trials of neoadjuvant immune checkpoint inhibitors in hepatocellular carcinoma.

NCT Number	Study name	Phase	Interventions	Status
NCT03510871	Nivolumab Plus Ipilimumab as Neoadjuvant Therapy for Hepatocellular Carcinoma (HCC)	2	Nivolumab + Ipilimumab	Recruiting
NCT05137899	Neoadjuvant Combination of Atezolizumab /Bevacizumab Versus Neoadjuvant Radiation Therapy	2	Atezolizumab+Bevacizumab vs Radiation Therapy	Not yet recruiting
NCT03630640	Neoadjuvant and Adjuvant Nivolumab in HCC Patients Treated by Electroporation	2	Nivolumab	Recruiting
NCT04727307	Neoadjuvant Atezo, Adjuvant Atezo + Beva Combined With RF Ablation of Small HCC: a Multicenter Randomized Phase II Trial	2	Atezolizumab	Recruiting
NCT03867370	Toripalimab in Combination With Lenvatinib as Neoadjuvant Therapy in Resectable Hepatocellular Carcinoma	1、2	Toripalimab + Lenvatinib	Recruiting
NCT04850040	A Neoadjuvant Hepatocellular Carcinoma Study of Camrelizumab in Combination With Apatinib and Oxaliplatin	2	Camrelizumab+Apatinib + Oxaliplatin	Not yet recruiting

NCT03299946	Feasibility and Efficacy of Neoadjuvant Cabozantinib Plus Nivolumab (CaboNivo) Followed by Definitive Resection for Patients With Locally Advanced Hepatocellular Carcinoma (HCC)	1	Nivolumab+ Cabozantinib	Active, not recruiting
NCT04615143	Tislelizumab Neo-adjuvant Treatment for Resectable RHCC	1	Tislelizumab	Recruiting
NCT04174781	Neoadjuvant Therapy for Hepatocellular Carcinoma	2	Sintilimab + TACE	Recruiting
NCT04196465	Phase II Study of Neoadjuvant Immune Checkpoint Inhibitor in Patients With Resectable Gastrointestinal Cancers	2	IMC-001(anti PD-L1)	Recruiting
NCT03337841	Pembrolizumab as Neoadjuvant Treatment in HCC	2	Pembrolizumab	Unknown status
NCT04930315	SHR-1210 Combined With Apatinib Mesylate in the Perioperative Therapy for Hepatocellular Carcinoma	2	Camrelizumab+Apatinib	Recruiting
NCT04850157	Tislelizumab Combined With IMRT Neoadjuvant Treatment for Resectable Hepatocellular Carcinoma With PVTT	2	Tislelizumab+ IMRT	Not yet recruiting

NCT03916627	Neoadjuvant Cemiplimab for the Treatment of Resectable NSCLC, HCC, and HNSCC	2	Cemiplimab	Recruiting
NCT04224480	Longitudinal Immune- phenotyping of HCC Following MK-3475	1	Pembrolizumab	Recruiting
NCT04888546	TQB2450 Combined With Anlotinib Hydrochloride in the Perioperative Treatment of Hepatocellular Carcinoma Hydrochloride Neoadjuvant Therapy for Resectable Hepatocellular Carcinoma With a High Risk of Recurrence or Metastasis	1、2	TQB2450(anti PD-L1) + Anlotinib	Recruiting
NCT04425226	Pembrolizumab and LENvatinib in Participants With Hepatocellular Carcinoma (HCC) Before Liver Transplant	Not Applicable	Pembrolizumab+Lenvatinib	Recruiting
NCT04123379	Neoadjuvant Nivolumab With CCR2/5-inhibitor or Anti-IL-8) for Non-small Cell Lung Cancer (NSCLC) or Hepatocellular Carcinoma (HCC)	2	Nivolumab+ BMS-813160/ BMS-986253	Recruiting
NCT04297202	SHR-1210 Combined With Apatinib Mesylate in the Perioperative Treatment of Hepatocellular Carcinoma	2	Camrelizumab+Apatinib	Recruiting
NCT04857684	SBRT + Atezolizumab + Bevacizumab in Resectable HCC	Early Phase1	Atezolizumab +Bevacizumab +SBRT	Not yet recruiting

NCT04521153	Camrelizumab Combined With Apatinib Mesylate for Perioperative Treatment of Resectable Hepatocellular Carcinoma	Not Applicable	Camrelizumab+Apatinib	Recruiting
NCT04658147	Feasibility and Efficacy of Perioperative Nivolumab With or Without Relatlimab for Patients With Potentially Resectable Hepatocellular Carcinoma (HCC)	1	Nivolumab ± Relatlimab	Recruiting
NCT04954339	DYNAmic Immune Microenvironment of HCC Treated With atezolIzumab Plus bevaCizumab	2	Atezolizumab +Bevacizumab	Recruiting
NCT04653389	Perioperative Therapy for Hepatocellular Carcinoma	2	Sintilimab+ TACE/ radiotherapy	Recruiting
NCT04443322	Durvalumab and Lenvatinib in Participants With Locally Advanced and Metastatic Hepatocellular Carcinoma	Not Applicable	Durvalumab+ Lenvatinib	Recruiting
NCT03222076	Nivolumab With or Without Ipilimumab in Treating Patients With Resectable Liver Cancer	2	Nivolumab ±Ipilimumab	Active, not recruiting

Table S2. Sensitivity analysis for Grade 3-4 TRAEs outcomes

	Grade 3-4 TRAEs			
	OR	95% CI	p-value	I^2
all	0.26	0.14-0.50	<0.0001	50%
Excluding Su Y et al	0.22	0.12-0.39	<0.00001	21%

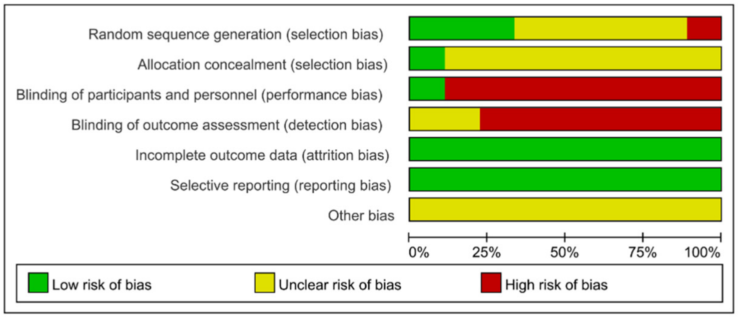

Figure S1. Risk of bias graph: review authors' judgment about each risk of bias item presented as percentages across all included studies.

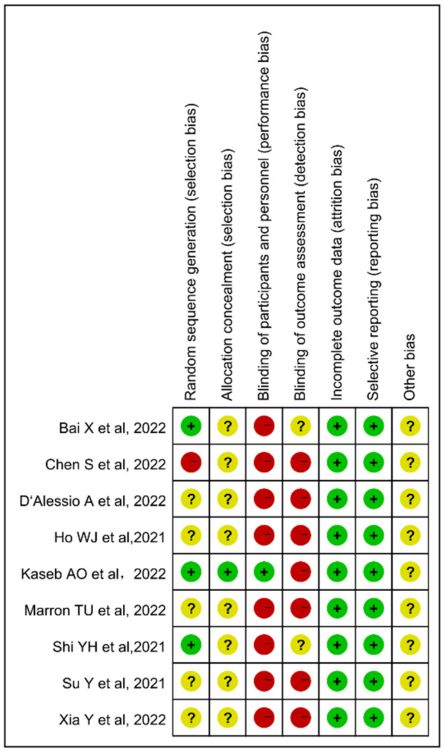

Figure S2. Risk of bias summary: review authors' judgments about each risk of bias item for each included study.
